# Terahertz spectral imaging based quantitative determination of spatial distribution of plant leaf constituents

**DOI:** 10.1186/s13007-019-0492-y

**Published:** 2019-09-13

**Authors:** Ziyi Zang, Jie Wang, Hong-Liang Cui, Shihan Yan

**Affiliations:** 1 College of Instrumentation and Electrical Engineering, Jilin University, Changchun, 130061 Jilin China; 2 Chongqing Institute of Green and Intelligent Technology, Chinese Academy of Science, Chongqing, 400714 China

**Keywords:** Terahertz imaging, Quantitative analysis, Plant leaf, Water content, Solid matter content, Gas content, Spatial variability, Plant disease detection

## Abstract

**Background:**

Plant leaves have heterogeneous structures composed of spatially variable distribution of liquid, solid, and gaseous matter. Such contents and distribution characteristics correlate with the leaf vigor and phylogenic traits. Recently, terahertz (THz) techniques have been proved to access leaf water content and spatial heterogeneity distribution information, but the solid matter content and gas network information were usually ignored, even though they also affect the THz dielectric function of the leaf.

**Results:**

A particle swarm optimization algorithm is employed for a one-off quantitative assay of spatial variability distribution of the leaf compositions from THz data, based on an extended Landau–Lifshitz–Looyenga model, and experimentally verified using *Bougainvillea spectabilis* leaves. A good agreement is demonstrated for water and solid matter contents between the THz-based method and the gravimetric analysis. In particular, the THz-based method shows good sensitivity to fine-grained differences of leaf growth and development stages. Furthermore, such subtle features as damages and wounds in leaf could be discovered through THz detection and comparison regarding spatial heterogeneity of component contents.

**Conclusions:**

This THz imaging method provides quantitative assay of the leaf constituent contents with the spatial distribution feature, which has the potential for applications in crop disease diagnosis and farmland cultivation management.

## Background

Plant leaves, composed of water, solid matter, and gas, play a key role in photosynthesis [1, 2], respiration [3] and water transport [4, 5]. The water abundance of leaves is closely connected with the vigor and phylogenic traits such as the structure, shape and photosynthetic efficiency of plants [6]. In addition, solid matter distribution and gas transport network are also linked with the dynamic metabolic activity in either raw material or product based on the formula of photosynthesis and respiration [7, 8]. Usually, abnormal concentration and distribution characteristics of constituent substances indicate the lack of nutrients or pests and diseases attack. Consequently, qualitative and quantitative methods for assessing spatial variability of water, solid matter and gas contribute to our understanding of plant response to environmental changes under normal and stress conditions [9, 10], which are indispensable for managing agricultural production. Thereinto, it is worth emphasizing that quantitative evaluation would play a more important role in establishing unified measurement standards, facilitating cross comparison between different samples and different species, and reflecting the state of plants more conveniently and accurately [11, 12], including leaf transpiration kinetics, plant water stress, and dry matter accumulation.

A number of techniques have been used to test the leave’s components quantitatively and provide technical support for precision agriculture. Gravimetry is the standard method for quantifying water contents of leaves by comparing the difference between the fresh and fully dried weights of the leaf. Gravimetric method is simple and reliable, but is destructive and non-real-time, unsuitable for noninvasive and continuous monitoring in greenhouses or fields. To avoid the disadvantage of destructive measures, more and more nondestructive testing (NDT) methods based on various parts of the electromagnetic spectrum have been used to develop agricultural sensing technologies [13]. The leaf water content of plant could be quickly and nondestructively detected through infrared [14], microwave [15], nuclear magnetic resonance [16], thermal imaging [17], and hyperspectral imaging [18], with the advantages of being label-free and in vivo, meeting the real-time and in situ monitoring requirements, such as water flow dynamics monitoring, temporal heterogeneity and spatial heterogeneity determination. Individually or jointly, these NDT techniques provide powerful support in the field of plant physiology and agronomy.

Meanwhile, a developing NDT technique based on the terahertz (THz) spectral region has shown great potential for water detection in the research fields of botany and agronomy [19, 20]. It is because that THz wave, extending from approximately 0.1 THz to 10 THz, coincides with the low-frequency vibration of the hydrogen bond, and is vested the high sensitivity to water molecules. Also, its low photon energy is soft enough for samples to avoid radiation damage. With the increasing ability of analyzing spectral information, the water content testing method based on THz spectroscopy has been maturing after undergoing three developmental stages, i.e., qualitative analysis, relative quantitative analysis and absolute quantitative analysis [21–23]. Unfortunately, almost all studies have overlooked the analysis of solid content other than water content, giving up on relevant information included in the spectrum due to the skeletal vibrations between or within nucleic acids, proteins, sugars and lipids, all falling within the THz range. In fact, the Landau–Lifshitz–Looyenga model (LLL model) [24] has the ability to calculate target contents by relating the dielectric permittivity of a heterogeneous mixture with the dielectric permittivity of its components. However, to date it has only been used to determinate leaf water content [23, 25], while the potential of comprehensive analysis of leaf component contents needs to be ascertained and realized, which would help differentiate the plant growth and development stages [26] and understand what parts of the leaf are the most sensitive at different metabolic states or under changing environmental conditions. Furthermore, nearly all of the quantitative analytical methods are based on a single-point spectral measurement, such as, establishing the line equation between water content and spectral parameters [27, 28], and calculating water content using an effective medium model [25, 29]. Such approaches would neglect the spatial heterogeneity of leaves, which is precisely the foundation for dissecting the metabolic processes of different leaf areas and tissues. More quantitative details about the spectral, temporal, and spatial variability of leaf contents by using graphical display could offer decision makers with intuitive and useful information about plants and crops.

In this article, we present a THz spectral imaging method to quantify the volumetric fraction of water, solid matter, and gas in leaf. In this method, the dielectric relationship between leaf and each component is given by an effective medium model, and the particle swarm optimization algorithm is introduced to calculate the relevant parameters. Our quantitative imaging results of leaves in different water states are strongly correlated with the traditional method, demonstrating the feasibility to monitor the spatial variability of leaf water, solid-matter and gas contents quantitatively using this new method, which are linked to the physiological characteristics of crops under conditions of damage, pests and disease. This study could help expand the application scenarios of terahertz spectral imaging in the field of plant physiology and agronomy.

## Materials and methods

### Sample preparation

**Fig. 1:**
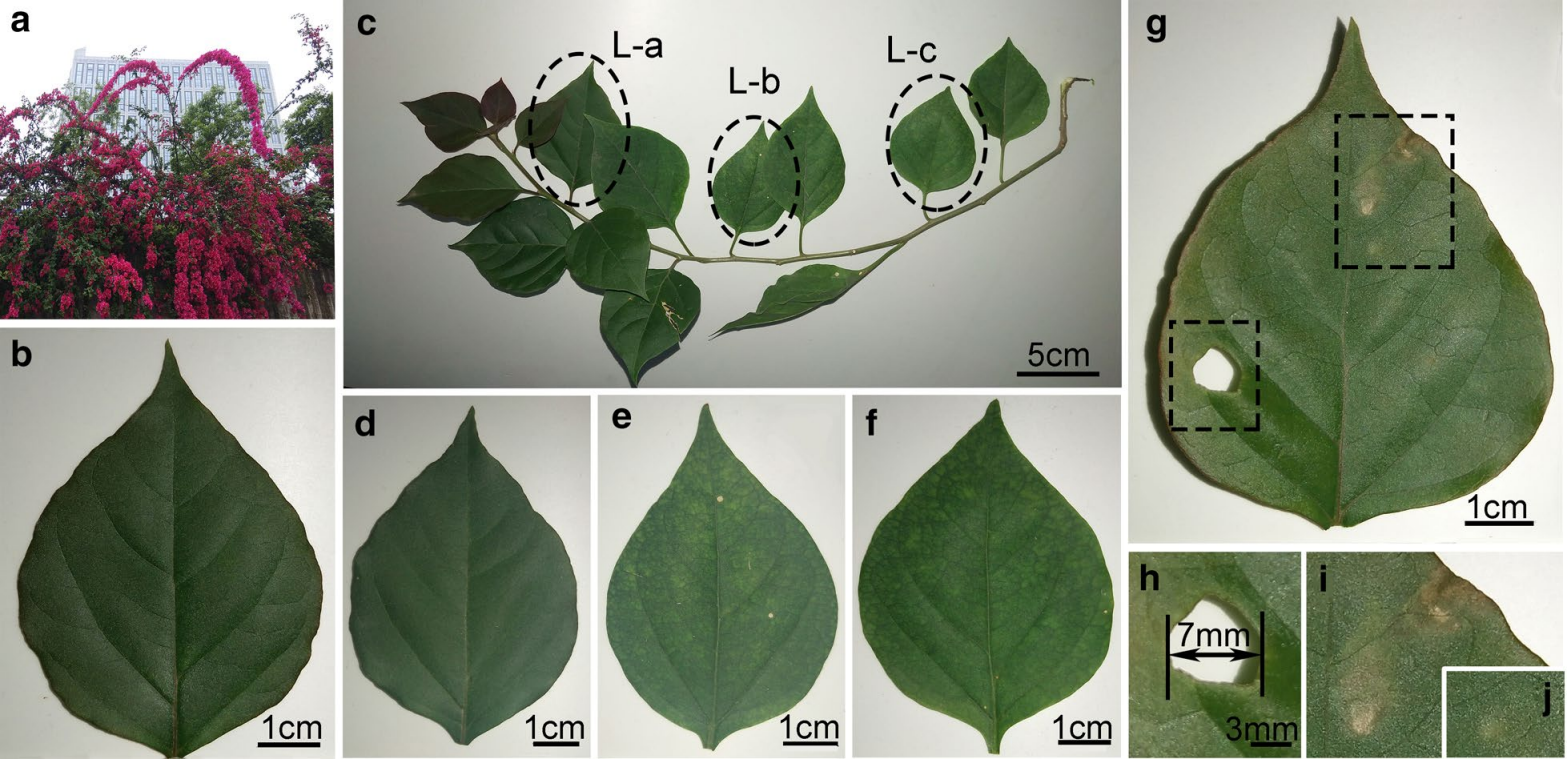
Photos of *Bougainvillea spectabilis* samples. **a** *Bougainvillea spectabilis* used for picking leaves. **b** Typical leaf. **c** Single branch of *Bougainvillea spectabilis*. Three leaves in a black circle from left to right were used for subsequent testing, corresponding to **d**–**f**, respectively. **g** Typical damaged leaf with a perforation (**h**) and some yellow mottling (**i**, **j**). Bars in **b**, **d**–**g** = 1 cm; **c** = 5 cm; **h**–**j** = 3 mm

About 80 individual whole leaf samples were picked from *Bougainvillea spectabilis* for this study, which were cultivated (Fig. 1a) on the campus of Chongqing Institute of Green and Intelligent Technology, Chinese Academy of Sciences, in Chongqing, China. Among them, more than 50 leaves randomly selected from different plants were used to establish the THz-based method. Besides, six sets of leaves, growing in different parts of a branch, from different plants were collected for investigating the sensitivity and test extremity of this method due to the subtle differences in water content of leaves at different maturity level. Further, five wounded leaves were selected to validate the ability of the method to detect special structures in leaves, especially for the suspected cases caused by pests and diseases. Figure 1b–i show the photos of representative leaves. All leaf samples were first used for THz spectral imaging, and then evaluated by gravimetric method.

For establishing the calculation method of distinguishing water, solid matter and gas components directly from the spectra of leaf complex, the individual solid matter and water need to be collected and tested firstly. Solid matter of leaves was prepared from the freeze-dried leaves by using the freeze dryer (SCIENTZ-30ND, Ningbo Scientz Biotechnology Co., Ltd., CN) and then pressed into compact dry-matter wafers about 300 microns thick by a small manual tablet press (HY-12, Tianjin Tianguang Optical Instrument Co., Ltd., CN). While, the pure water meets the grade III standard of Chinese laboratory water was obtained from a Reagent Water System (CLW-K10, Chongqing Qianlai Instrument Co., Ltd., CN).

### THz-TDS system and optical parameter extraction

**Fig. 2:**
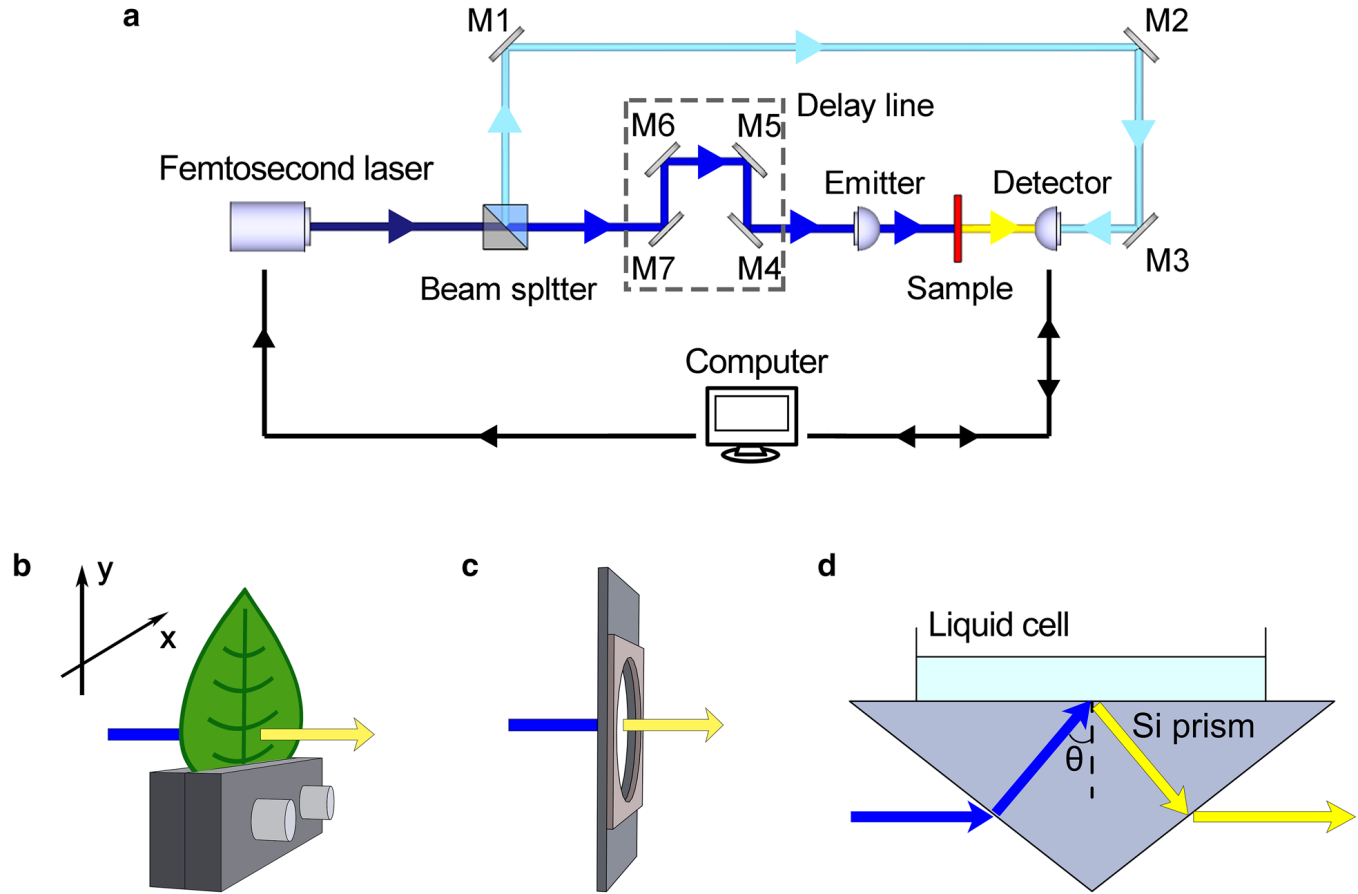
Schematic diagrams of a typical THz-TDS setup (**a**), and sketch of supporting holders, including XY two dimensional moving stage (**b**), solid sample cell (**c**) and ATR geometry (**d**)

A terahertz time-domain spectroscopic (THz-TDS) system (T-Gauge 5000 model from Advanced Photonix, Inc., USA) was deployed in our research, with a spectral bandwidth ranging from 0.1 to 3.5 THz, and a signal to noise ratio better than 60 dB at the frequency of analysis. The schematic diagram of the whole system in the transmission mode is displayed in Fig. 2a, and the XY two-dimensional moving stage was assembled into the system for imaging, shown in Fig. 2b. The raster scanning step size is 0.25 mm and the scanning speed is 50 mm/s, i.e., the dwell time at each pixel is 5 ms, longer than the spectral scanning time of 1 ms of the THz system. The imaging area of the XY-stage is 4.5 cm × 5.5 cm. The spatial resolution of the THz imaging system is about 1 mm. All the main specifications of the system meet the test requirements [30, 31].

For a sample with a rough surface, like leaves, the attenuation of electromagnetic waves results from both absorption and scattering, which means the contributions of absorption and scattering loss need to be considered, i.e., the total absorption coefficient $\upalpha =\upalpha_{\text{abs}} +\upalpha_{\text{scat}}$. A Rayleigh roughness factor was employed to describe the influence of scattering due to surface roughness [32]. The scattering-induced absorption was given by:

1 $$ {\text{a}}_{\text{scat}} = \left( {\Delta {\text{n}}\left( {\text{f}} \right)\frac{{4\uppi \uptau   {\text{cos}}\left(\uptheta \right)}}{\uplambda}} \right)^{2} \times \frac{1}{\text{d}} $$

2 $$ \Delta {\text{n}}\left( {\text{f}} \right) = \sqrt {\upvarepsilon_{\text{L}} \left( {\text{f}} \right)} - 1 $$

here, $\uptau$ denotes the degree of surface roughness expressed by the standard deviation of the height profile, $\uptheta$ is the angle of incidence, $\uplambda$ is the free-space wavelength, and d is the thickness of the leaf. Considering the scattering effect will lead to a more accurate leaves’ absorption coefficient.

The solid matter was tested in an iron square holder with a round hole, Fig. 2c, whose refractive index and absorption coefficient in THz band can be obtained by [33]

3 $$ {\text{n}}\left(\upomega \right) = \frac{{{\varphi }\left(\upomega \right){\text{c}}}}{{\upomega{\text{d}}}} + 1 $$

4 $$ \upalpha\left(\upomega \right) = - \frac{2}{\text{d}}{ \ln }\left( {{\text{A}}\left(\upomega \right)\frac{{\left[ {{\text{n}}\left(\upomega \right) + 1} \right]^{2} }}{{4{\text{n}}\left(\upomega \right)}}} \right) $$

where ${\text{A}}\left(\upomega \right)$ is the amplitude ratio of the Fourier transforms of the electric field transmission of the solution sample ${\text{E}}_{\text{s}}$ and the reference (the blank sample cell) ${\text{E}}_{\text{ref}}$, ${\varphi }\left(\upomega \right)$ is the phase difference between ${\text{E}}_{\text{s}}$ and ${\text{E}}_{\text{ref}}$, c is the velocity of light, and *d* is the thickness of the sample.

The optical parameters of water were obtained by attenuated total reflectance (ATR), using a setup that is mainly composed of a silicon prism with a refractive index of 3.42 in the THz band, shown in Fig. 2d. The THz pulse incident onto the ATR system is totally reflected at the interface between the prism and the sample, where an evanescent field is created on the sample side of the interface that interact with the sample. In THz-ATR spectroscopy, complex refractive index of liquid sample can be determined with higher accuracy than in commonly used reflection or transmission modes of THz-TDS [34, 35]. Assuming incidence from the prism side with the complex refractive index of $\widetilde{\text{n}}_{\text{pri}}$ to the sample side with the complex refractive index of $\widetilde{\text{n}}_{\text{sam}}$, with the incident angle $\uptheta$, $\widetilde{\text{n}}_{\text{sam}}$ is given by the solution of the simultaneous equations

5 $$ {\text{A}}\left(\upomega \right) = \left| {\frac{\text{r}}{{\text{r}^{\prime}}}} \right|^{2} $$

6 $$ {\varphi }\left(\upomega \right) = {\text{Arg}}\left[ {\frac{\text{r}}{{\text{r}^{\prime}}}} \right] $$

7 $$ {\text{r}} = \frac{{\widetilde{\text{n}}_{\text{sam}} { \cos }\uptheta - \widetilde{\text{n}}_{\text{pri}} \sqrt {1 - \left( {\frac{{\widetilde{\text{n}}_{\text{pri}} }}{{\widetilde{\text{n}}_{\text{sam}} }}{ \sin }\uptheta} \right)^{2} } }}{{\widetilde{\text{n}}_{\text{sam}} { \cos }\uptheta + \widetilde{\text{n}}_{\text{pri}} \sqrt {1 - \left( {\frac{{\widetilde{\text{n}}_{\text{pri}} }}{{\widetilde{\text{n}}_{\text{sam}} }}{ \sin }\uptheta} \right)^{2} } }} $$

where ${\text{r}}$ and $\text{r}^{\prime}$ are the Fresnel’s reflection coefficient of the prism-sample interface and of prism-air. The complex refractive index is $\widetilde{\text{n}} = {\text{n}} + {\text{ik}}$, and ${\text{k}} = {\text{c}}\upalpha\left(\upomega \right)/\left( {2\upomega} \right)$ is the extinction coefficient. And the real and imaginary parts of the complex dielectric constant are respectively

8 $$ \upvarepsilon^{\prime} = [{\text{n}}\left(\upomega \right)]^{2} - \left[ {{\text{k}}\left(\upomega \right)} \right]^{2} $$

9 $$ \upvarepsilon^{\prime\prime} = 2{\text{n}}\left(\upomega \right){\text{k}}\left(\upomega \right) $$

All the measurements were carried out in an environment with a constant temperature during the whole process (22 °C ± 0.1 °C). And the leaves were placed in ambient air during THz imaging, while the humidity was kept under 2% by dry nitrogen (N_2_) purge when measuring liquid and solid matter.

### Calculation of water, solid matter and gas contents

Effective medium approximation is an appropriate model to obtain the terahertz dielectric function of hydrated tissue. When the leaf is regarded as a combination of water, solid matter and gas, the leaf’s effective permittivity will be related with the permittivity and volume fractions of each component. Conversely, if the permittivity of leaf, ‘pure’ water, solid matter and gas are individually known, an effective medium model could be used to calculate their respective volume fractions.

Within a third-order extension of the LLL model proposed by Jördens et al. [29] to calculate leaf water content the dielectric function of the leaf is given by

10 $$ \sqrt[3]{{\upvarepsilon_{\text{L}} \left( {\text{f}} \right)}} = {\text{a}}_{\text{W}} \sqrt[3]{{\upvarepsilon_{\text{W}} \left( {\text{f}} \right)}} + {\text{a}}_{\text{S}} \sqrt[3]{{\upvarepsilon_{\text{S}} \left( {\text{f}} \right)}} + {\text{a}}_{\text{G}} \sqrt[3]{{\upvarepsilon_{\text{G}} \left( {\text{f}} \right)}} $$

where $\upvarepsilon_{\text{i}}$ are the dielectric functions and ${\text{a}}_{\text{i}}$ are the relative volumetric concentrations of the different components; the indices refer to leaf (L), water (W), solid matter (S) and gas (G) respectively.

**Fig. 3:**
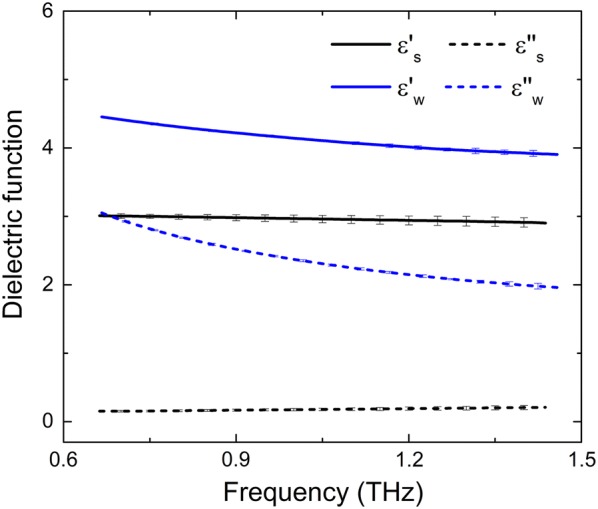
The real ($\upvarepsilon^{\prime}$) and imaginary ($\upvarepsilon^{\prime\prime}$) parts of the dielectric functions of solid matter ($\upvarepsilon_{\text{s}}$, black lines) and water ($\varepsilon_{\text{w}}$, blue lines) measured by THz-TDS and THz-ATR, respectively

As stated in the previous section, the dielectric function of the solid matter, shown on Fig. 3 (black lines), was determined by measuring compact dried *B. spectabilis* leaf wafers using THz-TDS; and the closed numerical value of different kinds of plants were detected due to the similar material composition (Additional file 1: Fig. S1). The dielectric function of deionized water determined by THz-ATR is shown on Fig. 3 (blue lines), in complete agreement with previously reported data [36]. The dielectric function of solid matter and water were used as inputs for the effective dielectric model.

A theoretical value of the dielectric function of leaf could be calculated by Eq. (10). Furthermore, Eqs. (3, 4, 8 and 9) are used to calculate a theoretical transmission coefficient of leaf ${\text{T}}_{\text{theo}}$. At the same time, the transmission coefficient of leaf ${\text{T}}_{ \exp }$ can be determined by THz-TDS. The coefficients ${\text{a}}_{\text{i}}$ that match ${\text{T}}_{\text{theo}}$ and ${\text{T}}_{ \exp }$ can be considered as the true volume fractions of each component of the leaf.

In order to match the theoretical transmission coefficient of leaf with that of the experimentally obtained, particle swarm optimization (PSO) algorithm with soft boundary condition has been adapted to adjusts the parameters ${\text{a}}_{\text{W}}$, ${\text{a}}_{\text{S}}$ and ${\text{a}}_{\text{G}}$. This algorithm is easy to implement and able to generate feasible solutions within a manageable amount of computation time.

Particle swarm optimization is a population-based iterative optimization technique that locates the solution to an optimization problem by allowing candidate particles to fly around the solution space. The candidate particles’ trajectories are affected by the best performing candidate solution (Gb) and the best location they have visited (Pb) [37]. The movements of the particles in canonical PSO are described by the following equations

11 $$ {\text{v}}_{{{\text{i}},{\text{k}}}}^{{{\text{t}} + 1}} =\upomega{\text{v}}_{{{\text{i}},{\text{k}}}}^{\text{t}} + {\text{c}}_{1} {\text{r}}_{1} \left( {{\text{p}}_{{{\text{Gb}},{\text{i}}}}^{\text{t}} - {\text{p}}_{{{\text{i}},{\text{k}}}}^{\text{t}} } \right) + {\text{c}}_{2} {\text{r}}_{2} \left( {{\text{p}}_{{{\text{Pb}},{\text{i}}}}^{\text{t}} - {\text{p}}_{{{\text{i}},{\text{k}}}}^{\text{t}} } \right) $$

12 $$ {\text{p}}_{{{\text{i}},{\text{k}}}}^{{{\text{t}} + 1}} = {\text{p}}_{{{\text{i}},{\text{k}}}}^{\text{t}} + {\text{v}}_{{{\text{i}},{\text{k}}}}^{{{\text{t}} + 1}} $$

where ${\text{v}}_{{{\text{i}},{\text{k}}}} \left( {{\text{i}} = {\text{water and solid matter}}} \right)$ and ${\text{p}}_{{{\text{i}},{\text{k}}}} \left( {{\text{i}} = {\text{water and solid matter}}} \right)$ are the velocity and position of the ${\text{kth}}$ particle; $\upomega$ is the inertial weight; the constants ${\text{c}}_{1}$ and ${\text{c}}_{2}$ are acceleration coefficients; ${\text{r}}$ is a uniform random number within [0,1].

The particle positions and velocities are randomly initialized. Afterwards, they move in the solution space guided by Eqs. (11, 12). Once it goes beyond the boundary, the particle will be reset to its previous position and its flight path will be reprogrammed. The fitness of all particles are evaluated and the global and personal best positions are updated if needed. The global best at the end of the simulation is taken as the solution to the problem, which is the volumetric fraction of water, solid matter and water in the present case.

The convergence reliability of PSO for solving percentage volume is the fundamental element for quantifying the distribution map of each component. Therefore, 10 THz spectra were randomly selected from 50 leaves’ THz imaging data sets and 30 calculations were performed independently on each spectral data to verify the performance of PSO applied in quantitative analysis of the leaf’s three-component model. Table 1 shows a typical set of the results; the relative standard deviation of the calculated result of each component content is less than 5%, and in particular, that of water less than 1%. This fact indicates that the content of each component of the leaves obtained by the PSO calculation is statistically trustworthy.

**Table 1:** Results of particle swarm optimization in solving the leaf’s three-component model

	Content			
	Mean (%)	Maximum (%)	Minimum (%)	Relative standard deviation (%)
Water	74.2	74.3	74.1	0.1
Solid matter	20.0	20.2	19.1	1.6
Gas	6.0	6.5	5.8	3.9

### Terahertz spectral imaging for quantitative distribution map

Point-by-point THz spectral imaging was performed on the tested leaf by THz-TDS and the two-dimensional moving stage for collecting spectral data of each point on the leaf. The imaging data were then processed by a software program written in MATLAB which integrates the LLL model and PSO algorithm to calculate the water, solid matter and gas content. The program could recombine single point data to draw distribution maps of water, solid matter and gas content. These results were stored as a data group and displayed visually through the images.

### Gravimetric water content testing

To evaluate the accuracy of the THz measurements, each leaf was stored in a plastic bag directly after imaging, and the fresh weight (FW) was determined by using an electronic balance (ME204E, Mettler-Toledo, CH) immediately afterward. After drying the leaves at 95 °C for 4 to 8 h until the weight is constant, the corresponding dry weights (DW) were determined. The gravimetric water content (GWC) and the gravimetric solid content (GSC) were calculated for each leaf using the following equations [38, 39]:

13 $$ {\text{GWC}}\left( {\text{\% }} \right) = \frac{{{\text{FW}}\left( {\text{g}} \right) - {\text{DW}}\left( {\text{g}} \right)}}{{{\text{FW}}\left( {\text{g}} \right)}} \times 100{\text{\% }} $$

14 $$ {\text{GSC}}\left( {\text{\% }} \right) = \frac{{{\text{DW}}\left( {\text{g}} \right)}}{{{\text{FW}}\left( {\text{g}} \right)}} \times 100{\text{\% }} $$

## Results

### The establishment and adequation of the method for the quantitative assessment of spatial variability of water, solid tissues and gas in plant leaf

**Fig. 4:**
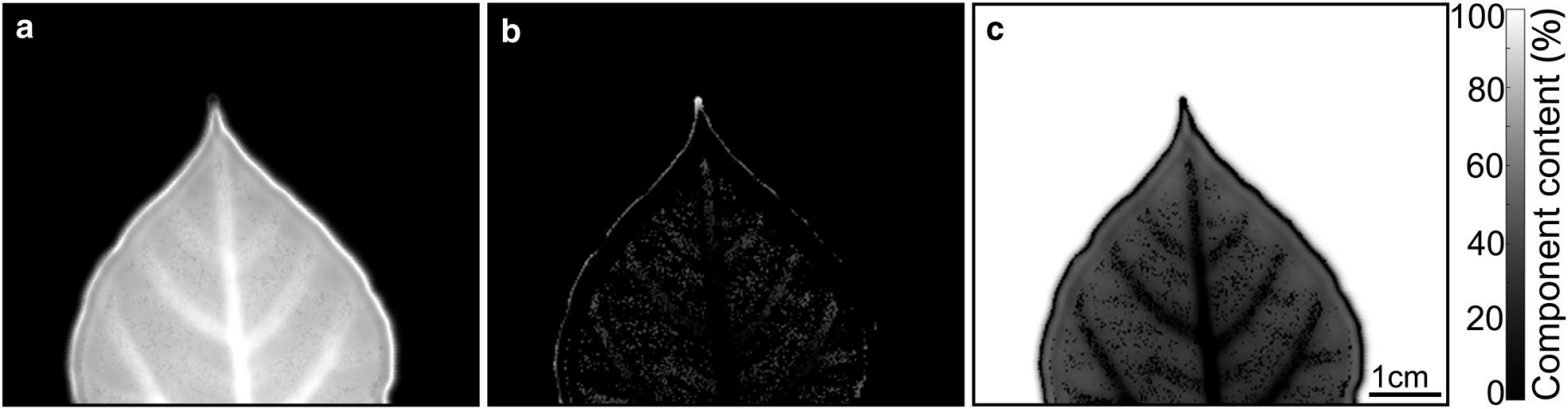
THz images showing leaf water (**a**), solid matter (**b**), and gas (**c**) content. The color bar indicates the actual content value of each component, with a range from 0% (total absence, black) to 100% (total presence, white). Scale bar in **a**–**c** = 1 cm

By collecting the THz transmission spectral data and processing them with the LLL model and PSO, the leaf (Fig. 1b) 2-D images were constituted (Fig. 4) and each pixel (0.25 mm by 0.25 mm in size) in the image corresponds to the percentage volume of water, solid matter or air on one certain point of the leaf. The color bar represents the exact values of percentage volume and a lighter color matches a higher material content. These three images respectively reflect the spatial distribution of water (Fig. 4a), solid matter (b), and gas (c) in the leaf, and show the spatial positions of veins and mesophyll. In general, there is more water, less solid matter and less air in veins.

**Fig. 5:**
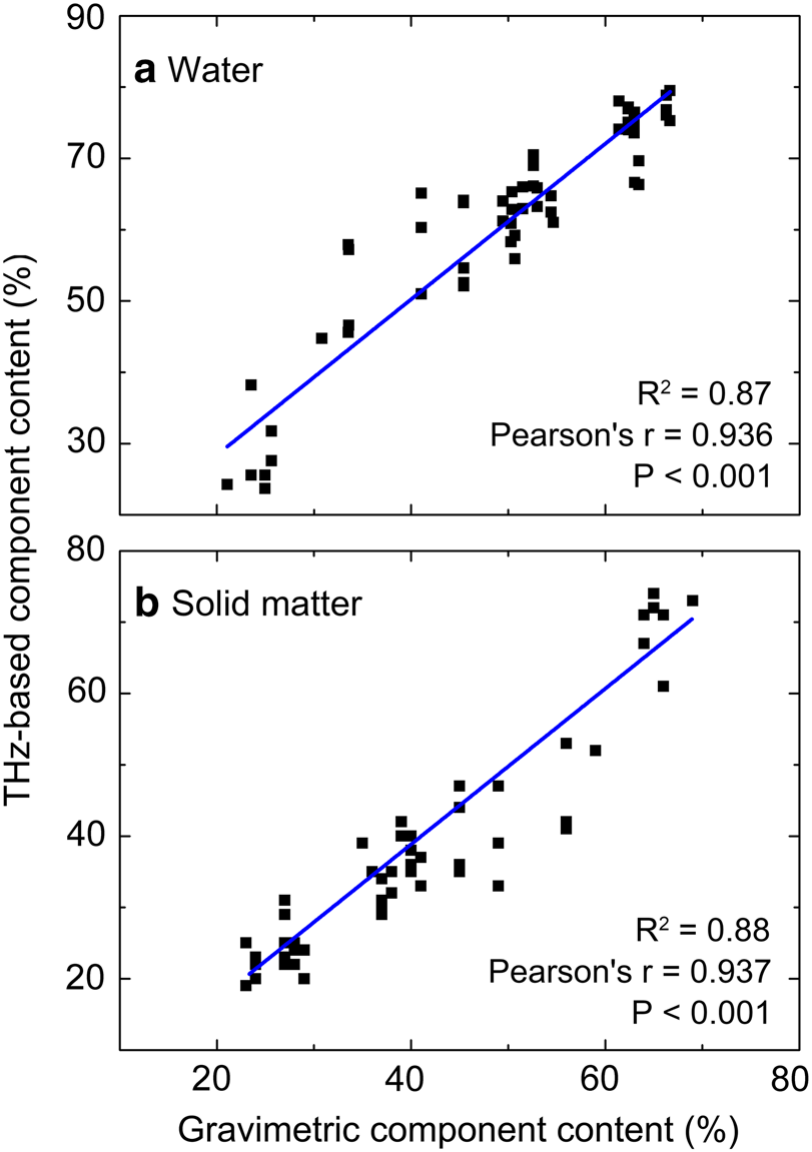
Correlation analysis of THz-based method and weight-based method. The Pearson correlation coefficients of water (**a**) and solid matter (**b**) are 0.936 and 0.937 respectively; and the R^2^ values are 0.87 and 0.88 respectively

Next, the leaf water and solid matter contents were measured through the traditional gravimetric method to further corroborate the reliability and applicability of the THz-based method. A group of leaf disks with different matter contents, made from the blade stripped of main veins and cut into rectangles of about 2 cm^2^ with different natural air-drying times, were used for both THz imaging and weight-based water content determination. For terahertz imaging, the average of corresponding parameters is used to characterize the substance content state of the whole leaf. From Pearson analysis results, the correlation coefficients of water (Fig. 5a) and solid matter (Fig. 5b) are 0.936 and 0.937. And the coefficient of determination of water and solid matter are 0.87 and 0.88 respectively. The results indicate that there is a strong linear positive correlation between THz-based measurement and the gravimetric one, which demonstrated that the algorithm proposed in this paper converges to the correct constituent substances proportions for the leaf, and the quantitative analysis results based on the THz signals are credible.

### Sensitivity of THz spectral discrimination

**Fig. 6:**
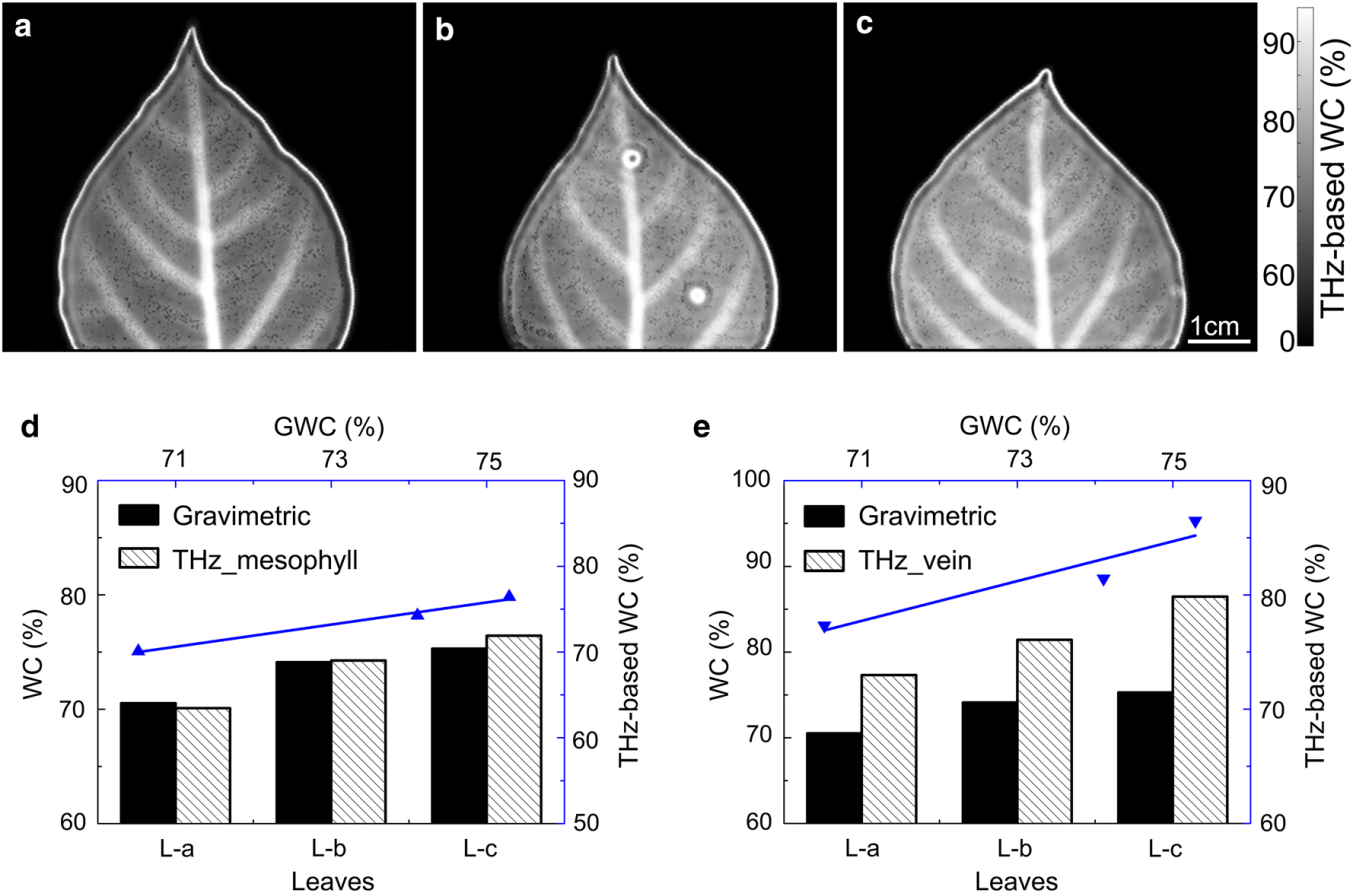
**a**–**c** Water content maps of three leaves, L-a (**a**), L-b (**b**) and L-c (**c**) located at the tip (Fig. 1d), middle (Fig. 1e) and root (Fig. 1f) of the same branch, based on THz technology. The color bar indicates the actual value of THz-based WC, and scale bar in **a**–**c** = 1 cm. **d**, **e** Contents (histogram, black) and correlation analysis (blue line) of water content of mesophyll (**d**) and vein (**e**) measured by THz-based method and the leaf water content measured by gravimetric method, where R^2^ values are 0.99 and 0.89 respectively. In mesophyll, THz-based WC = 1.291 × GWC − 0.211, while in lateral vein, THz-based WC = 1.739 × GWC − 0.457

Water accounts for the largest proportion in leaf constituents and is vital to the healthy growth of plants. Under normal physiological conditions, the dynamic range of water content in leaves is limited [40], requiring a sufficiently sensitive test method for predicting material changes. Water content is the most representative index for testing the sensitivity of a given quantitative assessment. Three whole leaf blades located in the same branch, as shown in Fig. 1c, were comparatively analyzed with the THz method and the weighing method. The water content of L-a (at the tip of the branch, shown in Fig. 1d), L-b (at the middle of the branch, shown in Fig. 1e), and L-c (near the trunk, shown in Fig. 1f) are 70.52%, 74.11%, and 75.29%, respectively, measured by gravimetric method. THz images were used to quantitatively describe subtle distinction or variation among leaves at different maturity level. During the process, the contrast ratio of THz images was enhanced by stretching the gray value of 0.6–0.9 in order to improve the differentiation of water content visually. From Fig. 6a–c, the brightness of the grayscale image gradually increased, indicating that the water content of the leaves increases gradually from top to base of the branch, consistent with the trend of the results tested by the traditional method. This agreement is demonstrated more clearly by exact numerical values extracted from THz images, shown in Fig. 6d and e, where the average THz-based water content of the mesophyll region are 70.09%, 74.26% and 76.44%, while that of the lateral veins are 77.32%, 81.43% and 86.48%. These tests proved that THz-based water content measurement method has sufficient sensitivity to quantitatively detect the water content difference of leaves in similar states.

### THz leaf wound marking based on changes in composition and structure

**Fig. 7:**
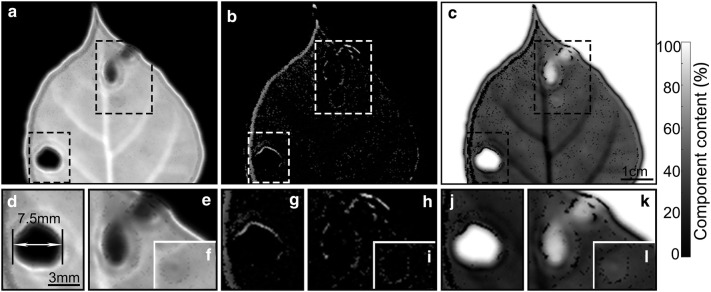
THz images of a damaged leaf (Fig. 1g) based on water (**a**), solid matter (**b**), and gas (**c**) content. There is a perforation in the lower left side and some yellow mottling in the upper right side of the leaf (Fig. 1h–j). **d**–**i** Magnification of the damage details. The color represents the actual value of component content, and scale bars in **a**–**c** = 1 cm; **d**–**i** = 3 mm

Water, solid matter and gas as fundamental chemical constituents in plants formed vegetative tissues together, and individual content abundance is closely influenced by the leaf vigor, and abnormal morphological characteristics always indicate the lack of nutrients or the damage by pests and diseases. In this study, several types of leaf wounds, including a perforation and some yellow spots in one naturally damaged leaf (Fig. 1g, f) are clearly presented through THz spectral imaging (Fig. 7). These different kinds of foliar injuries are displayed clearly in these images based on the spatial variability of water (Fig. 7a), solid matter (b), and gas (c). The predictable and obvious lower water, lower solid matter and higher gas content to penetrating injury are detected in the THz images, and the numerical value is the same as the blank background. Further, the number, positions and shapes of yellow spots at different stages are also confirmed through the THz image features, whereas not all these connected spots with different degrees damage could be exactly detected in the optical images. Compared to penetrating injury, the gray values of the two upper yellow spots are higher in water-based and solid matter-based images, and lower in gas-based image, while compared to the normal blade locations the spots in water- and solid matter-based THz images are darker, and lighter in a gas-based image. Besides, the lighter areas around the wound in Fig. 7b, g–i show the higher solid matter content. From the above, the ability of THz imaging to describe the wounds and other special structure in leaves has been revealed.

## Discussion

The water-based image (Fig. 4a) and gas-based image (Fig. 4c) show clear outline of veins, while the differences between veins and mesophyll are somewhat cloudy in the solid matter-based image. Veins are penetrated with vascular tissue and have special channels for water transport, thus containing more water than mesophyll [16], resulting in the significant contrast between vein and mesophyll in water-based image. On the other hand, the gas transport network in leaf was mainly composed of the intercellular air space through the mesophyll. Gas molecules permeate mesophyll due to the abundant tissue with large interstitial space. But the wall cells that make up the catheter and sieve tubes in veins are densely arranged and the extracellular matrix increased collagenic and fibrous contents in the wall, impeding gas exchange and circulation [41]. These morphological features could be reflected in gas-based image. The blade section stained images of a serials of different kinds of dicotyledonous plants, with dyeing nuclei, chromosomes and plant proteins, could be used to help ascertain that the anatomy and morphology characteristics between vein and mesophyll were indeed different, but solid matter content alone exhibited too little difference to act as a marker [42]. Moreover, the similarity of the THz transmission signals at the top of the main vein, secondary veins and mesophyll after natural drying also indicated a similar solid matter content between veins and mesophyll [31], consistent with the solid matter-based image (Fig. 4b). In addition, it should be pointed out that the bright and dark streaks at the leaf margin are due to diffraction, which has nothing to do with the structure of the leaf itself, and it does not affect the internal details of the images. Thus we observe that the THz imaging results suggest that the proposed method can be used to image leaf morphology and has the potential to detect the spatial variability of the water, dry-mater and gaseous-matter contents of plant leaves.

Gravimetry reflects the average water distribution in leaf including mesophyll and veins. The mesophyll water content based on THz imaging is closer to the gravimetric one (Fig. 6d) due to the higher proportion of mesophyll in leaf. And the fact that the water content in veins is higher than that in mesophyll determines [16] that the measurement value from THz imaging in veins is greater than weighing result (Fig. 6e). The results from these two methods cannot be perfectly matched, but the variation trend from the THz imaging was consistent with the gold standard for the water content measurements. Moreover, our analysis revealed another interesting phenomenon that there was a greater difference in the values of water content measured by the two methods in leaves farther away from the tip of the branch. It is known that the leaf water content actually consists of both free water and bound moisture (hydration water) [43]. And the absorption of bound water in the THz band is larger than that of free water [44]. When the difference between free water and hydration water was ignored during the calculation, the water volume percentage would appear to be higher than the actual value, and the error would increase with the increase of the bound moisture content. The tender leaf in the tip had more free water due to the faster metabolic rate in cells, resulting in the smaller difference. Nuances in the results such as this seem to indicate that the new method based on THz spectral imaging has greater potential to divide the intracellular water into free water and bound moisture, which proportions are strongly associated with metabolic levels [45].

In addition, a potential application of wound detection has been proposed because the grayscale characteristics in THz images of yellow spots are different from those of normal mesophyll and penetrating injury (Fig. 7). Compared to the normal blade the solid matter contents in the gangrene affected regions which were under conditions of environmental stress and/or insect pests and plant disease is lower [46] but still existed. The increase of dry matter around the wound shown in the THz images may come from the accumulation of lignin, callous, and such, which can help slow down the water loss and improve the disease resistance of the plant [47–49]. Besides, the thickness of the leaf was overestimated at the wound, as the leaf was considered to be uniform during the calculation process. Consequently the calculated value of gas content was higher, as the gas content near the wound surface was incorrectly included. More noteworthy is the fact that this developing method based on THz imaging determined the boundary cleanly and clearly through different indicators in the earlier stage when the optical instrument was not able to define the scope of the yellow leaf spot. This development of sensitive THz imaging platforms and precise image processing schemes has opened up the possibility toward disease diagnostic studies based on water, solid matter and gas variation of plant leaves. These results heralded the capability of THz imaging for early warning of plant blade damage, and the potential to identify disease types, because the substance compositions and morphological characteristics of plant lesion tissues vary greatly with different disease types and development degrees [50].

## Conclusions

A THz spectral imaging method combining a particle swarm optimization based on an extended Landau–Lifshitz–Looyenga model was proposed and demonstrated, which could visualize water, solid matters and gas quantitative distributions in the leaves. Good agreement between the THz-based measurement results and standard gravimetric data suggested that the THz detection method has a huge potential to measure the leaf material contents in a simple, fast, non-destructive, and label-free manner. Details of component content and morphological structure in different blade parts could be detected clearly through the leaf THz images, which indicated that the THz imaging would play an important role in the field of crop disease diagnosis and farmland cultivation management. With the continuous development and improvement of the theory and technology of THz spectroscopy and imaging methods, it could be expected to become a standard tool in the research field of botany, agronomy, and crop science in the near future.

## Supplementary information

**Additional file 1: Fig. S1.** Dielectric permittivity of water and three kinds of leaves’ solid matter. The three kinds of solid matter from leaves have similar values of real part (black lines) and imaginary part (blue lines) of dielectric permittivity, which are much smaller than those of water.

## Data Availability

The raw data from all experiments as well as the material used in this manuscript can be obtained from the corresponding author upon reasonable request.

## Abbreviations

ATR: attenuated total reflectance
DW: dry weights
FW: fresh weight
GWC: gravimetric water content
GSC: gravimetric solid content
LLL model: Landau–Lifshitz–Looyenga model
NDT: nondestructive testing
PSO: particle swarm optimization
THz: terahertz
THz-TDS: terahertz time-domain spectroscopic
WC: water content
