# Supplementary figures and images for: Terahertz spectral imaging based quantitative determination of spatial distribution of plant leaf constituents
Source: Plant Methods. 2019 Sep 13;15:106. doi: 10.1186/s13007-019-0492-y (PMC6743168; doi:10.1186/s13007-019-0492-y)

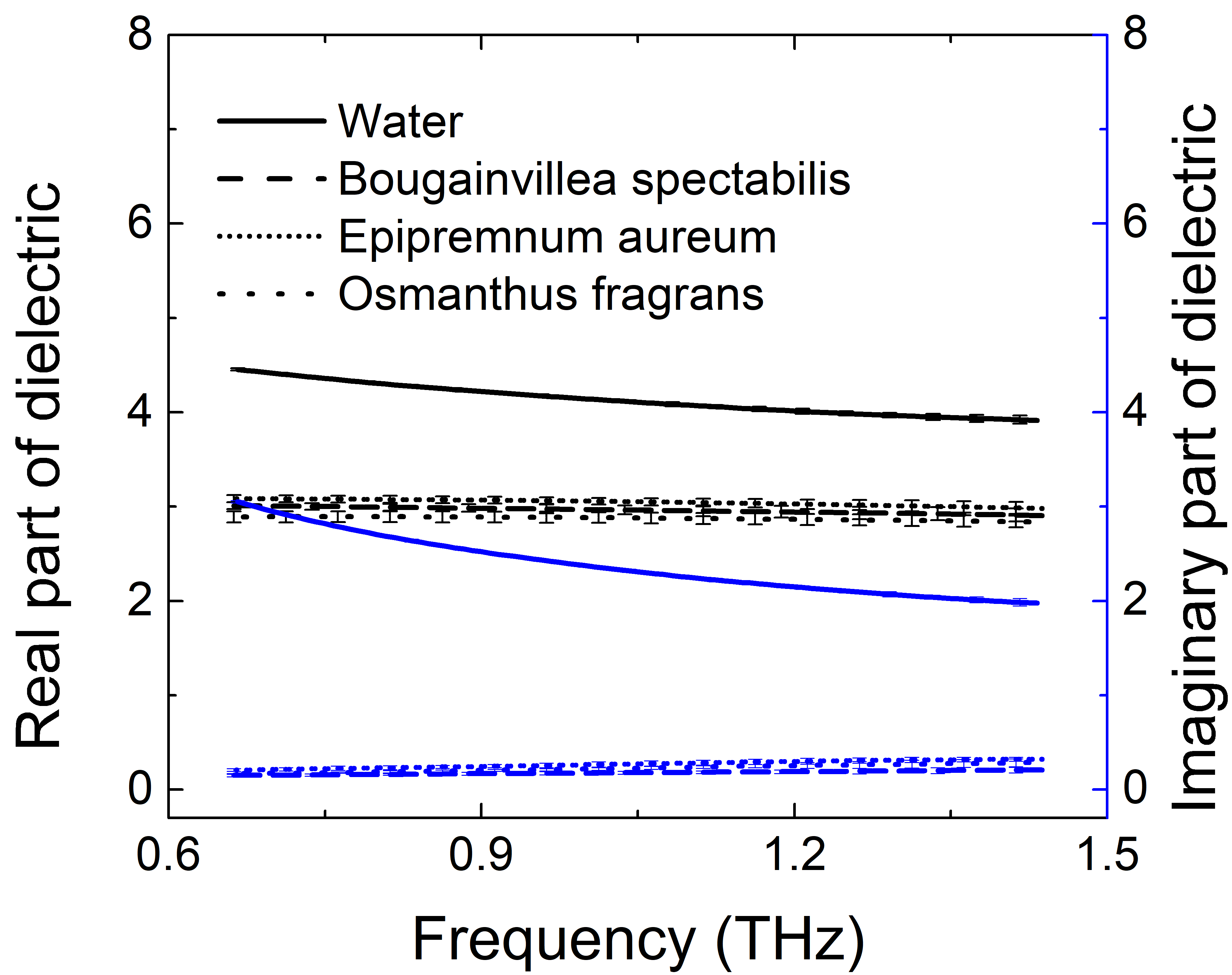

Supplement: Supplementary file 1 — Additional file 1: Fig. S1. Dielectric permittivity of water and three kinds of leaves’ solid matter. The three kinds of solid matter from leaves have similar values of real part (black lines) and imaginary part (blue lines) of dielectric permittivity, which are much smaller than those of water. [file 13007_2019_492_MOESM1_ESM.tif]
